# Legacy in End-of-Life Care: A Concept Analysis

**DOI:** 10.3390/nursrep14030177

**Published:** 2024-09-14

**Authors:** Carolina Timóteo, Joel Vitorino, Amira Mohammed Ali, Carlos Laranjeira

**Affiliations:** 1 Urgency Department, Local Health Unit of the Leiria Region—Hospital of Santo André, Rua das Olhalvas, 2410-197 Leiria, Portugal; carolina.timoteo97@gmail.com; 2 School of Health Sciences, Polytechnic University of Leiria, Campus 2, Morro do Lena, Alto do Vieiro, Apartado 4137, 2411-901 Leiria, Portugal; joel.vitorino@ipleiria.pt; 3 Palliative Care Unit, Portuguese Institute of Oncology of Coimbra, 3000-075 Coimbra, Portugal; 4 Department of Psychiatric Nursing and Mental Health, Faculty of Nursing, Alexandria University, Smouha, Alexandria 21527, Egypt; amira.mohali@alexu.edu.eg; 5 Centre for Innovative Care and Health Technology (ciTechCare), Polytechnic University of Leiria, Campus 5, Rua das Olhalvas, 2414-016 Leiria, Portugal; 6 Comprehensive Health Research Centre (CHRC), University of Évora, 7000-801 Évora, Portugal

**Keywords:** legacy, end of life, concept analysis, death, dignity

## Abstract

Comprehending the significance of legacy in end-of-life (EoL) situations helps palliative care professionals enhance person-centered outcomes for those with a life-threatening illness and their families. Our purpose was to conduct a concept analysis of legacy in EoL care. By employing Walker and Avant’s approach, we identified the concept’s defining characteristics. Subsequently, we established the antecedents, consequences, and empirical referents. After conducting a thorough review of titles and abstracts, a total of 30 publications were analyzed. These articles were sourced from three databases (CINAHL, Medline via PubMed, and Scopus) from 2002 to 2023. Our analysis identified several core attributes of legacy: (a) leave behind something of value that transcends death; (b) determine how people want to be remembered; (c) build and bestow across generations; (d) integrate advance care planning through EoL conversations and shared decision-making; and (e) develop strategies of dignity-conserving care. The consequences are related to improvements in spiritual and subjective well-being; coping with inevitable EoL existential issues; decreases in EoL suffering; engendering self-awareness, hope, gratitude, and peace; achieving and maintaining dignity; creating good memories; promoting mutually constructive and transformative relationships; and fostering the adjustment of bereaved people. Nevertheless, further effort is required to implement the key attributes of legacy that form the basis for creating legacy-oriented interventions near the EoL.

## 1. Introduction

In their relationship with illness and the approach of death, patients at the end of life (EoL) “inhabit” what Sinclair [1] calls the liminal space between life and death. What we are and how we “inhabit” this space and this time of life and death does not belong just to the field of direct and real relationships with the disease but rather to the totality of our being in all its dimensions. Simply put, the entire human person is shaped not only by the physical and palpable relationship with the real world but also by spirituality, lived experiences, learning, and each person’s inner experience. From these multiple interactions, an internal repertoire is built that is capable of guiding each person in responding to opportunities, crises, and, ultimately, the destabilization that the disease brings—the Personal Resources of Dignity [2]. Thus, human suffering and the individual capacity to manage it can only be understood through the appreciation of resources, the personal repertoire of dignity of each person at the EoL [3]. 

The protective perspectives of dignity refer to each person’s internal qualities, personality, and personal vision of the world (i.e., the way of looking at one’s life situation). These perspectives reflect each person’s internal characteristics and their evolution when confronted with death [4]. By creating a legacy, in its various forms, human beings deny the cessation, stagnation, or vital ineffectiveness of life, trying to ensure that their example and their existence can convert the world into a better place to live [5]. A legacy represents in itself the firm desire to accomplish something that survives one’s own death. By sharing something of themselves with others, the human person materializes their gift, expanding their shared love in the present, relinquishing their intimacy and isolation [6,7], projecting themselves into future generations. For the person at the EoL with a reduced prognosis, existential challenges become unsettling, and the issue of legacy, of continuing beyond the announced end, becomes an inevitable objective to be fulfilled [8]. For these patients, the question “What meaning does my life have now?” evolves into restlessness: “What difference will my life make after I leave?”

In the Dignity Model proposed by Chochinov et al. [6,7], the elaboration of a legacy (generativity) describes the feeling of comfort, consolation, and purpose felt by patients when leaving something lasting and transcendent after their own death. The EoL is overcome through the feeling of continuity and personal appreciation. Many patients intuitively stimulate their dignity, creating a verbal narrative of their lives, leaving a legacy of their deeds and contributions, enabling an eternal connection with those they will leave behind [9]. Promoting legacy as a psychotherapeutic intervention responds to the psychosocial and existential anguish of patients at the EoL, inviting them to report and discuss the life issues that are most important to them, articulating them so they are remembered after the approaching death [10]. These discussions and memories can be audio recorded, transcribed, and edited into a final legacy document that can be delivered to family members or other loved ones [11]. The legacy that survives in the consciousness of all who receive it is a tangible form of durability beyond death.

Cicely Saunders, the pioneer of the contemporary hospice movement, emphasized the need to listen to the individual’s story in order to deliver high-quality care and facilitate a peaceful death. The starting point is to consistently perceive the patient as an individual and, therefore, as someone who possesses cognitive abilities, emotions, interpretation, social skills, and creativity, and it has the potential for continuous growth throughout their lifetime, even if the future offers restricted opportunities [12]. In this vein, a person-centered palliative care involves a meaningful dialogue with the seriously ill person to acquire a deeper understanding of their living circumstances [13].

With adult patients, the feeling of lack of meaning, usefulness, and purpose in life can constitute a serious threat to their dignity, especially if this is not limited to the present but spreads to the future of others [14]. In this way, a brief psychotherapeutic intervention based on the concept of legacy/generativity could create something that transcends death, that is, something that faithfully represents who people were at the end of their lives and what they felt and expressed when they thought about who they loved [15].

Assuming that legacies are made, experienced, and shared throughout a variety of developmental periods, pediatric palliative care understands legacy as a precious instrument of support for parents of children at the EoL as a way of reducing their suffering. Parents of children with incurable diseases are encouraged to make an impression of their child’s hand or a plaster cast of their foot as a way of physically and symbolically “eternalizing” emotional memories [16,17]. In these cases, more than the legacy object itself, what is also important is the process, the ritual of creation and the deep connection it holds for each party involved [18]. Thus, the legacy transcends its material meaning and gains an unattainable and profound dimension perceived and experienced by those familiar with it [19].

Prior research suggests that there are three distinct categories of legacies: primary, secondary, and tertiary [20]. Primary legacy comprises the artifacts that a deceased person deliberately bequeaths and their desires regarding how they wish to be remembered. This may encompass a diverse range of material and social artifacts, including but not limited to financial and legal records, professional merchandise, and mementos designed to preserve the memories and social standing of cherished individuals [21]. For individuals living with severe illness [22], legacy planning can serve as a rite of passage or a process of adaptation. It may also involve making decisions regarding medical care. Secondary legacy is how bereavement and memorialization activities [23] are initiated by others in remembrance of a deceased loved one or family member. The implementation of social distancing regulations during the COVID-19 pandemic led to significant changes in memorialization practices. Unfortunately, because of these changes, those who experienced loss during this period were more likely to develop prolonged grief disorder [24]. Lastly, tertiary legacy refers to acknowledging the professional, political, or international influence of a public figure who the memorialized does not necessarily know personally [25].

To the best of our knowledge, there is no conceptual analysis that elucidates how individuals confronted with severe illness perceive their legacy. Evidence also suggests that perceptions of legacy at the EoL are highly idiosyncratic [18], creating vagueness in its conceptualization and building. In fact, without a consensual definition of “legacy”, the practice, training, and quality of palliative care are also compromised, namely the ability to offer a person-centered care approach. In addition, Boles and Jones [26], in their foundational contribution about legacy interventions for children and adults receiving palliative care, suggest the need to clarify the borders of the legacy concept. Therefore, the purpose of this analysis is (1) to guide the development of an operational definition of legacy in EoL care and (2) to identify attributes, antecedents, and consequences of the concept of legacy to enhance the understanding of the concept and its implications for palliative care.

## 2. Materials and Methods

### 2.1. Study Design

This concept analysis was conducted using the eight-step model proposed by Walker and Avant [27]. In the first step, the concept is selected; in the second, the purpose of the analysis is clarified; and in the third, an overview of all uses of the concept is identified. The third step includes a literature search of all usages of the term “legacy”. This process aids in the delineation of the concept’s defining attributes (step four). This is followed by an iterative process to develop model cases and additional cases (steps five and six). In step seven, the attributes, antecedents, and consequences of the concept are identified and refined. In the final step (eight), empirical referents are chosen to determine the defining attributes of the legacy concept [27].

### 2.2. Data Sources and Analysis

A comprehensive literature search was conducted using CINAHL, Medline via PubMed, and Scopus. The database research was conducted from March 2024 to May 2024. The search approach was adapted from the Medline database and subsequently implemented on other databases. A combination of keywords using Boolean operators and truncation (*) was utilized in the following search string: ([Title/Abstract] “legac*” OR [Title/Abstract] “life purpose” OR [Title/Abstract] “life history” OR [Title/Abstract] “life review” OR [Title/Abstract] “dignity” OR [Title/Abstract] “heritage” OR [Title/Abstract] “patrimony” OR [Title/Abstract] “inheritance” OR [Title/Abstract] “living legac*” OR [Title/Abstract] “existing legac*” OR [Title/Abstract] “wish*” OR [Title/Abstract] “memories”) AND ([Title/Abstract] “palliative care” OR [Title/Abstract] “end of life care” OR [Title/Abstract] “terminal care” OR [Title/Abstract] “hospice care” OR [Title/Abstract] “Hospice and Palliative Care Nursing”). 

Inclusion criteria comprised publications addressing legacy within EoL care; written in Portuguese, Spanish, and English; and with full-text availability. Only studies involving palliative care staff, informal caregivers, and adult patients eligible for or currently receiving palliative care were integrated. Exclusion criteria included the grey literature (i.e., unpublished dissertations, and thesis), editorials, and conference proceedings. No temporal filters were placed on the literature search.

Researchers collaboratively developed, filled out, and validated a centralized data excel spreadsheet. Two researchers (C.T and J.V.) handled all stages of study selection, appraisal, and data extraction. Any inconsistencies were discussed with a third researcher (C.L.) until a consensus was reached. Data were synthesized to define the characteristics, antecedents, and consequences using an integrative thematic analysis [28]. Then, the same or similar content was combined to compare and classify all extracted content until the concept’s features became clearer.

## 3. Results

**Figure 1:**
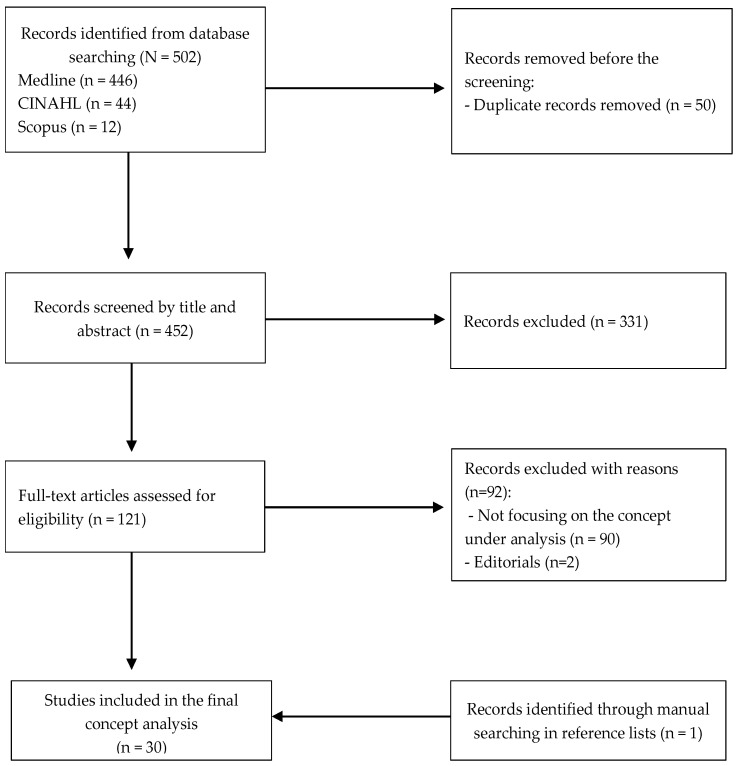
Flow diagram illustrating selection of sources.

A total of 502 publications were discovered in the databases using this method. The papers underwent verification for English-language publications, the availability of abstracts, and the presence of duplicate publications. During the initial screening, papers were considered if the search term appeared in the title and/or abstract. The full text of 121 articles was then analyzed. Of these, 92 articles were excluded because they did not respond to the aims of study or were editorials. One further article was obtained by examining the reference lists of the papers. In the end, 30 articles relating to the concept of legacy in EoL care were included in the concept analysis (Figure 1). All documents were published between 2002 and 2023 and were from the following countries: the United States (*N* = 7), Canada (*N* = 5), Australia (*N* = 4), China *(N* = 4), New Zealand (*N* = 2), Sweden (*N* = 2), the United Kingdom (*N* = 2), Germany (*N* = 2), Singapore (*N* = 1), and Japan (*N* = 1). The study designs were mainly qualitative (*N* = 16). Six publications were reviews, and there were two case studies and one discussion paper. A summary of the included publications is depicted in Supplementary Table S1.

### 3.1. Use of Term in Dictionary Definitions

Merriam–Webster online dictionary defines legacy (as a noun) as “something transmitted by or received from an ancestor or predecessor or from the past”. The term “legacy” also refers to “property bequeathed by a will” or a “gift left by a will” [29]. Similarly, Cambridge English Dictionary defines legacy as “something that is a part of your history or that remains from an earlier time” [30]. Additional etymological origins connote the term legacy as a specifically appointed representative, ambassador, envoy, or possibly a “messenger.” Upon analyzing these etymological roots, it becomes evident that “legacy” indeed fulfills the most crucial function as the conduit via which we pass on essential information, values, traditions, and wisdom to future generations.

### 3.2. Defining Attributes

According to Walker and Avant, attributes play a fundamental role in identifying different concepts. A concept may have more than one attribute, but it is necessary to define the concept’s best attribute [27]. Legacy is one avenue by which an individual may bring their life story to a close by effectively conveying essential aspects of their identity to future generations [31]. Viewing “legacy” as a means of passing on spiritual and cultural knowledge and wisdom implies a constantly ongoing process. This implies an act (planned or serendipitous) that is reserved for the anticipated or actual EoL, allowing for people to define how they would like to be remembered [31,32,33] by leaving behind something of value that transcends death [32,34,35,36,37,38,39,40,41], such as a life story, digital legacy (i.e., social media profiles, email accounts, photographs), writing, painting, or other forms form of tangible creations. Embedded in transgenerational dynamics [35,37,42], the legacy becomes a journey back in time, a narrative that unfolds over time. By recognizing and dealing with patterns that are passed down through generations, people and families can begin a process of profound growth and healing. According to O’Callaghan [43], legacy is a long-lasting manifestation of an individual’s characteristics, life experiences, impacts, and connections that is built and bestowed to subsequent generations.

Grewe [44] states that legacy has no negative implication when it comes to the desire to be remembered and having self-awareness. Legacy provides individuals with diverse reasons for creating something to leave behind. Rather than driven by altruism, it is based on mechanisms that can benefit both the individual and the receivers of activities aimed at creating something lasting or impactful [31,33]. To achieve that purpose, several strategies for dignity conservation and legacy have been implemented, such as resilience, a life review, reminiscence, and hope-based interventions [34,37,43,44,45,46].

The process of creating a legacy begins early in life and coexists with other motivations and desires. However, for individuals in the oldest age group or at the end of their lives, the act of leaving a legacy may assume a heightened significance. Advance care planning can result in improved care by facilitating a deeper comprehension of a patient’s desires and fostering stronger communication between patients and healthcare providers [47,48]. In this sense, healthcare personnel must possess a high level of education to ensure they are at ease and self-assured when participating in advance care planning sessions. This involves comprehending the potential influence that early advance care planning can have on creating a legacy [47,48]. In this vein, legacy should become part of advance care planning conversations based on honesty, authenticity, informed participation, and shared decision-making [44,48]. Effective communication and strong relationships between the care team, patient, and family are crucial factors in promoting legacy in EoL care. This tone of care allows for the patient or their family to express the patient’s care preferences while affirming that they genuinely matter [32].

### 3.3. Constructed Cases

The subsequent phase of a concept analysis involves the construction of a case model as well as the identification of borderline and contrary cases [27]. This study utilized a model case that was derived from real-world examples, encompassing all the concept’s essential attributes. Borderline cases are examples that contain most of the defining attributes but not all of them. Contrary cases contain none of the defining attributes [27]. Despite the use of real-life examples, ethical approval was unnecessary for this study as it did not involve gathering private identifiable information or patient consent.

#### 3.3.1. Case Model

M.A., female 74 years-old, is from a typical village on the coast of Portugal, where she has lived since she was 54, after having emigrated to Luxembourg for 30 years. About 8 years ago she was diagnosed with breast carcinoma and underwent surgery and chemotherapy. In March of last year, a recurrence was diagnosed, and a liver metastasis was identified, with no curative viability. Due to clinical deterioration, she was referred to a palliative care unit in the center of the country.

The physical deterioration and loss of functionality were evident in the first week, with M.A. expressing a desire to participate actively in defining her care plan as well as in planning the farewell rituals, which according to her should be respected. She expressed her desire to be accompanied in this end-of-life process by J.A., her lifelong companion and someone she trusted to ensure that her wishes were met and her beliefs respected. She would like to wear the typical costumes of her village at her funeral as a way of demonstrating the importance she attached to maintaining her people’s customs and traditions and as a symbol of identity that she would like to pass on to subsequent generations, especially her grandchildren. Realizing that J.A. was suffering as he faced the end of her life, M.A. asked the health team to help minimize the impact of her death on her husband and help build and convey a message of comfort, resilience, and above all hope for the future after her death. The team intervened in the M.A.–J.A. dyad, promoting communication and the expression of emotions, seeking to integrate the loss by attributing meaning to their experiences. They also created a digital storytelling to share with the family, in which they reinforced the aspects they considered important to perpetuate in the family they had created: love, respect for others, solidarity, and altruism. 

After M.A.’s death, J.A. sent a letter of thanks to the medical/care service in which he stressed the importance of the activities he had carried out at the end of his wife’s life, which had allowed him to soothe her pain by respecting her wishes and honoring her life. The healthcare team also felt the impact of this intervention due to the inherent awareness and confrontation with finitude and its inherent consequences.

#### 3.3.2. Borderline Case

C.A., 72, a male patient with pancreatic cancer, who was admitted to a palliative care unit at the end of his life, expressed several concerns to the team, including his wish to die at home. The team operationalized his wish in advance care plan. 

His daughter (F.A.), 42, single, a family member who cared for him at home, had difficulty managing her emotions and viewed her father’s hospital discharge reluctantly, as she would not be able to meet her father’s care needs. The support of a palliative community team was limited by the high number of patient referrals and the existence of a waiting list. As C.A.’s clinical condition worsened, he expressed his wish to leave a digital letter to his daughter and grandchildren, in which he recounted the highlights of his life and the values that guided his decisions and choices. The care team helped him write the digital letter. Even though he was not at home, he felt at peace, as he was not a burden on his family. After his death, F.A. felt sad because she could not fulfil her father’s wishes and failed her role as daughter. 

#### 3.3.3. Contrary Case

M.R., a female Angolan national aged 74, was in Portugal visiting a sister when she was admitted to the emergency department with advanced chronic heart failure, severe edema, and severe dyspnea. Her symptoms prevented her from carrying out the basic and instrumental activities of daily living. 

Throughout her hospitalization, as she became aware of the rapid deterioration in her state of health, she repeatedly expressed her desire to return to her country so that she could die at home with her most significant family members. Given her weakened condition, the trip was inadvisable. 

M.R. developed anxiety in the last few days of her life. This was accompanied by motor agitation, which resulted in increased dyspnea, so she was submitted to palliative sedation. M.R. died without accomplishing her desire to return home. Her family, especially her children, showed signs of a higher risk of grief disorder prolonged by feelings of guilt, as they had not followed their mother’s situation at the end of her life. 

### 3.4. Antecedents

Antecedents come before defining attributes, and all of them must be present for the concept to be realized [27]. Evidence indicates that human beings have an inherent inclination to form and sustain social connections, even when death is impending [34,49]. While there may be idiosyncrasies, in general, humans possess a high level of sensitivity towards the viewpoints of others. Essentially, we are concerned about the opinions of others; we desire to be well liked and to be part of a community with which we feel a sense of belonging. In this sense, the desire to “leave” a legacy may stem from our inherent inclination as human beings to form and cherish meaningful connections with others and to be esteemed during our lifetime [31,32,36,40,43,48,50,51,52]. 

Although the inherent desire to develop intimate and emotionally reciprocating connections with people is widely recognized, legacy requires a sense of autonomy and self-determination [36,43] as well as self-identity [31]. Legacies encompass more than a mere sequence of events; they are situated inside a narrative framework that imparts significance to the human perception of time, space, and individual agency. Legacy is in fact spiritual and cultural DNA [37,53,54,55] that provides a mission and purpose leading to authentic and full lives.

Receiving legacy-oriented interventions or EoL care requires that professionals possess some key characteristics, such as sensitivity, empathy, interest, and presence [40]. Built on a relational foundation, healthcare providers must acknowledge the potential benefits of legacy interventions, such as fostering connection, self-expression, and community [49].

### 3.5. Consequences

According to Walker and Avant, consequences are events or results that occur after the concept [27]. Legacy building helps patients with symptom management (i.e., anxiety, depression, dyspnea) [44], leads to less suffering at the EoL [44,49,56], and, consequently, to spiritual and subjective well-being [35,38,48,51,57]. In this sense, creating a legacy helps patients deal with the inevitable existential questions at the EoL [44,49], allowing them to resolve unfinished business [52]. In parallel, creating legacy leads to good memories [43], promotes self-awareness [57,58], brings comfort [32], and fosters hope, gratitude, and peace [34,36,37,48]. In EoL care, legacy helps achieve and maintain dignity [34,36,37,48] in a mutually constructive and transformative relationship [44,55]. In turn, the memories linked to legacy might cultivate bonding connections with a deceased person. Legacy creation provides the bereaved with memories and physical objects that validate a life, helping to generate comforting connections with the deceased [35,43,44,51]. It also prepares people with a life-limiting illness for a future they will not be part of, helping them reflect on their life [40]. The concept of legacy can have a significant impact on the bereavement process [51,59] by facilitating a continued link between the bereaved and the deceased person as well as their community. Perpetuating memories through legacy-oriented interventions (i.e., digital legacy) [41,59] may facilitate the preservation of a relationship with a deceased person. This aligns with the theory of continuous connections, affirming that relationships undergo changes after death but persist rather than terminate. It suggests that maintaining a connection with a departed someone can be considered normal, adaptive, and reassuring.

### 3.6. Empirical Referents

In the last stage of this analysis, we established the practical indicators of the concept [27]. According to the literature review, evaluating the concept of legacy within EoL care is challenging without a standardized measurement tool. However, we identified three empirical referents with proximity to our core concept: the Zimbardo Time Perspective Inventory (ZTPI), the Dignity Care Intervention (DCI), and the Patient Dignity Question (PDQ). The ZTPI assesses an individual’s temporal orientations and capacity to be present in the current moment [60]. The DCI is a self-assessment instrument that enables professionals to facilitate dialogue and promote patient consciousness regarding care related to dignity [60]. It is based on the philosophy of person-centered care for meeting patients’ palliative care needs. Another tool that assesses patient priorities and stressors is the Patient Dignity Question (PDQ), “What do I need to know about you as a person to give you the best care possible?” [32]. It is a dignity-conserving intervention that serves as a meaningful EoL legacy document that benefits patients, staff, and families. Providing patients with the chance to articulate the impact of their condition enables healthcare providers to have a more profound comprehension of the specific requirements of each individual. The resulting written record can serve as a lasting legacy to be passed on to loved ones, thereby offering a significant “ultimate gift”.

### 3.7. Definition of Concept

After analyzing the available literature, we have formulated a concise definition of legacy concept that may be applied in the field of EoL care: Legacy serves as a unifying element that connects the past, present, and future, providing a sense of purpose in life. It implies the idea of having a life witnessed by others, whether planned or serendipitous. The goal is to generate memories or symbols/objects specifically crafted to elicit memories that will serve as a favorable encounter in the present and elicit positive emotions when recalled in the future. The singularity of each legacy emphasizes the need for a person-centered care approach that is able to respect human dignity, support relationality, and solve end-of-life existential issues.

**Figure 2:**
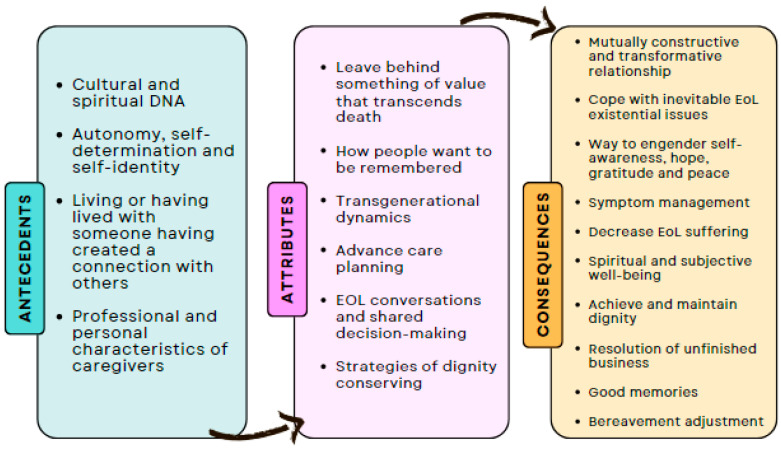
A conceptual diagram of legacy in EoL care.

Figure 2 illustrates a conceptual model that depicts the theoretical connection between the attributes that *explain* legacy in EoL care, its antecedents, and the resulting consequences.

## 4. Discussion

The concept of ‘legacy’ assumes that most people want to feel that their life had meaning and that this will somehow endure—a desire that becomes stronger as death approaches. The organization of the ‘legacy’, with the guidance of health professionals in palliative care, aims to respond to this need and assumes a therapeutic nature [47,61]. This is a practice that is little talked about, requires preparation from professionals, and is still uncommon in palliative care units—such as, for example, encouraging and helping the patient to gather in a box some of the objects they most appreciated in life, making an album with the most significant photographs, writing a letter or recording a video or audio statement evoking memories, and talking about ideas and people that are important in their life [24,47,61].

Palliative care is rooted in a humanistic life-valuing approach that respects people not only for their past, memories, and life story but also for their present state and potential accomplishments [62]. The goal of EoL care is to maintain an individual’s dignity by providing comfort, mitigating existential distress and addressing their various anticipated needs, which include physical, psychospiritual, social, and environmental aspects [63]. Attaining that purpose, people need to have their life witnessed and memorialized by others. Hence, palliative care teams must develop the necessary activities to create a ‘legacy’ for patients, as this practice is clearly in line with the central philosophical tenet of palliative care: “*You matter because you are you, and you matter to the last moment of your life*” [64]. As proposed by Harvey Chochinov, “intensive caring” provides a means for healthcare professionals to support patients who are facing significant levels of suffering [65]. Attempting to address inherent flaws in the healthcare system might lead healthcare professionals to have a sense of helplessness and failure. However, “intensive caring” offers a chance to concentrate on attainable objectives when helping patients feel that *they matter* [65]. Legacy-oriented interventions can be used to facilitate introspection and contemplation about one’s distinct attributes, aspirations, and influence on others [57]. These interventions can create a connection between patients and healthcare providers in situations of ambiguity, addressing the social, emotional, and spiritual requirements of patients and their families who are seeking or undergoing palliative care [23,66]. Likewise, there is increasing evidence that by creating their ‘legacy’, the patient also feels relief from the physical symptoms of the disease, which also contributes to validating this practice [67]. 

In an ever more virtual age, the significance of digital legacy has grown exponentially. There is empirical evidence indicating that the implementation of digital legacies in palliative care has a positive impact on the emotional well-being of patients and facilitates better communication [68]. Healthcare professionals should receive education on how to promote legacy to empower them to initiate interactions with patients and families as part of their ongoing legacy work and memory making interventions [69]. Further investigation is required to comprehend the full extent of the immediate and long-term effects that legacy interventions may have as well as to determine the most effective methods for addressing the needs of patients and their families in palliative care. Additional research is required to gain a comprehensive understanding of the effects of legacy on the bereavement journey. Instead of confining legacy to the creation of a final product in the face of death, this concept analysis points to the importance of adopting journey-oriented methods that prioritize death literacy and enable communities to support people who are dying and their carers. 

Various limitations arose during this concept analysis. Few studies directly incorporated legacy into their research. Likewise, we used a limited number of databases, limiting the extension of this concept analysis. The term “legacy” may initially be confined to specific linguistic traditions or cultural modes of thinking. Future research should further investigate the extent to which the concept of legacy can be transferred between different languages, cultures, and traditions. Considering the growing number of digital natives, more research is needed to explore the perspectives and experiences of patients and carers about digital legacy [59]. In addition, the effects of legacy-oriented interventions in all aspects of palliative care need to be investigated. Our understanding is currently limited, and it is crucial to increase the awareness and importance of legacy in both healthcare and society as a whole. 

## 5. Conclusions

This study’s antecedents, critical attributes, and consequences of legacy in EoL care offer a necessary basis for the provision of dignity-conserving care to those dealing with life-limiting conditions and guiding future research and practice in palliative care. A legacy is an intangible manifestation of the transient connections and interactions associated with a person, place, or time. It encompasses a tone of care that fosters non-abandonment, brings hope, and aids personal growth and fulfillment. This concept analysis highlights how legacy serves as a remembrance element across time and helps providers support EoL patients and their families in coping with distress and achieving better adjustment to situational demands. By having this comprehension, providers may more effectively respect the distinct individuality, connections, and abilities of people in the most desperate situations. When professionals advocate for legacy, they can broaden their therapeutic imagination to encompass the potential for patients to experience psychological, spiritual, and physical relief; bearable pain; and, for those in their latter stages, a peaceful death.

## Data Availability

The data are available upon reasonable request. This article is based on the first author’s master’s dissertation in Palliative Care at the School of Health Sciences—Polytechnic University of Leiria.

## Supplementary Materials

The following supporting information can be downloaded at https://www.mdpi.com/article/10.3390/nursrep14030177/s1, Table S1: Characteristics of the studies included in the review (n = 30).

## Public Involvement Statement

There was no public involvement in any aspect of this study.

## Guidelines and Standards Statement

This manuscript was drafted against the eight-step model proposed by Walker and Avant [27].
