# Supplementary material for: Legacy in End-of-Life Care: A Concept Analysis
Source: Nurs Rep. 2024 Sep 14;14(3):2385–97. doi: 10.3390/nursrep14030177 (PMC11417748; doi:10.3390/nursrep14030177)
Supplement: Supplementary file 1 [file nursrep-14-00177-s001.zip › nursrep-3150729-supplementary.pdf]

Table S1. Characteristics of the studies included in the review (N=30)

Ref.	Author(s)/ Year / Country	Type of Study	Aims	Setting/ Participants	Key findings
31	(Keall et al., 2011)<br><br>Australia	Original paper<br><br>Qualitative study	The aim was to explore what perceptions people with life-limiting illnesses have of life (positive or negative), and what message they want to leave in the future, for the next generations.	Eleven participants were chosen, both in hospital and in palliative care, both inpatient and outpatient.	There were three common themes: life review, current situation, and legacy/principles. In palliative care, each patient has a sense of self, their view of the current situation, their hopes, and how they would like to be remembered.
32	(Chochinov, 2002)<br><br>Canada	Case Study	The aim was to identify whether patients can maintain a sense of dignity and whether demographic and disease-specific variables are related to dignity.	A patient, his wife and a physician.	Each patient and their family hold a distinct definition of dignity, which clinicians should recognize to deliver the most compassionate and holistic end-of-life care. The dignity model provides a structure for clinicians to approach this responsibility, making dying with dignity a deliberate objective. When dying individuals feel acknowledged and valued by their caregivers, the preservation of dignity is more probable. Furthermore, caregivers, by acting with dignity, are equipped to offer care and comfort to those approaching the end-of-life.
33	(Ent and Gergis, 2020)<br><br>USA	Original paper<br><br>Quantitative study	To identify the most common end-of-life nurses' reflections among terminally ill patients.	124 nurses from the Hospice and Palliative Nurses Association.	Common themes of these reflections included concern for loved ones, regret, morbidity and mortality, gratitude, spirituality, legacy, and thoughts about acceptance or non-acceptance of impending death. Nurses reported that their patients were more concerned about their loved ones than their own morbidity and mortality. Common end-of life reflections may serve as cues that hospice and palliative patients are self-initiating therapeutic life review.
34	(Harstäde et al., 2018)<br><br>Sweden	Review article	The study aimed to find suggestions from Swedish research literature on what kind of care actions can preserve dignity of patients in a palliative care.	An integrative literature review. Articles published from 2006 to 2015 and theses published from 2000 to 2015 were searched the terms 'dignity' and 'palliative care'.	The adapted Dignity Care Intervention is suggested as a way for Swedish nurses to provide person-centred palliative care that will conserve patients' dignity. Suggested care actions included listening, communication, information, symptom control, facilitating daily living and including patients in decision-making. In addition, dignity-conserving perspectives includes continuity of self, maintenance of pride, generativity/legacy, hopefulness, acceptance, autonomy/control and resilience/fighting spirit.
35	(Von Post and Wagman, 2019)<br><br>Sweden	Review article	The aim was to describe what patients in palliative care describe as important at the end-of-life.	Seventeen articles were included in the review, and they were based on interviews.	The findings indicate that maintaining active involvement in one's occupation is crucial for individuals nearing the end-of-life. They prioritize the ability to sustain their established occupational routines and lifestyle to a significant degree. Five sub-themes emerged: maintaining previous occupational patterns; feeling needed; being involved in the social environment; leaving a legacy; and living as long as you live. Occupational therapists can contribute to this by taking a highly person-centered approach and gaining information about what matters most for their clients.

36	(Ho et al., 2013)<br>China	Original paper<br>Qualitative study	A study was carried out on the lived experience of Chinese patients and how their families received palliative care in Hong Kong, between the end of 2009 and the beginning of 2011.	18 Chinese terminal patients, aged 44 to 98.	Engaging in ritual practices that foster individual autonomy and strengthen family bonds is vital for supporting Chinese patients nearing the end of life. These actions play a key role in aiding patients to find comfort and meaning amid the challenges of mortality, all while upholding their dignity.
37	(Ho et al., 2013)<br>China	Original paper<br>Qualitative study	To examine the concept of 'living and dying with dignity' in the Chinese context and explore the generalisability of the Dignity Model to older terminal patients in Hong Kong.	16 older Chinese palliative care patients with terminal cancer.	The results emphasize the significance of both cultural and familial aspects in defining dignity. They stress the crucial need for cultural proficiency and understanding when interacting with diverse ethnic groups. Additionally, they advocate for an approach to palliative care interventions for elderly Chinese terminal patients that is both culturally sensitive and family-centered.
38	(Allen, 2009)<br>USA	Original paper<br>Qualitative study	Aims to examine the efficacy of a family-based dyadic intervention to decrease caregiving stress and increase family communication.	Seventeen individuals living with chronic, life limiting illnesses in the community. Their family caregivers received three home visits with a master's level interventionist.	All the participants in the intervention group started a Legacy Project and reported that the activities improved family communication. It also resulted in an increase in positive emotional experiences in the patient and carer groups.
39	(Johnson, 2007)<br>New Zealand	Original paper<br>Qualitative study	Seeks to elucidate the understanding of hope among patients facing terminal illnesses, aiming to establish hope as a nursing concept supported by evidence while prioritizing the quality of life for terminally ill patients in the days ahead.	17 pieces of research-based literature on hope.	Ten critical characteristics of hope were recognized: positive expectation; personal qualities; spirituality; goals; comfort; help/caring; interpersonal relationships; control; legacy; and life review. By the conclusion of the concept analysis, a definition and elucidation of the concept in its present application were attained.
40	(Collier, 2016)<br>Australia	Original paper<br>Qualitative study	The aim of this article is to explore the potential of video-reflexive ethnography as a practice development methodology to improve care of people with a life-limiting illness in the hospital setting.	Patients and families in hospital setting.	The research results underscore the capability of video-reflexive ethnography as a developmental method for practice. The use of video broadened its impact beyond the immediate research sites, helping to humanize the experience of hospital end-of-life care. The study served as a transformative innovation, highlighting the expertise of both patients, families, and healthcare workers. Additionally, for certain participants, the research provided a visual heritage.
41	(Welsch and Gottschling, 2021)<br>Germany	Review article	This article aims to provide an overview of the final matters that must be considered in the last phase of an adult patient's life.	204 articles analysed.	When caring for individuals with terminal illnesses, greater emphasis should be placed on managing the end-of-life phase. Palliative care specialists are suited to assume this responsibility from other medical fields, emphasizing the benefits of early integration into palliative care services. Most people want to discuss matters relating to the end of life with their physician in order to fulfil their last wishes (digital and emotional legacies).

42	(Bray et al., 2018)<br><br>New Zealand	Original paper<br><br>Qualitative study	This study aimed to explore the lived experience of migrants dying while away from their country of birth or origin.	The purposive sample of New Zealand immigrants at the end of life and under palliative care were interviewed at home.	Implications for end-of-life care include education to increase practitioner awareness and use of formal and informal life review. Enhancing spiritual well-being can assist resolution of end-of-life adjustment.
43	(O'Callaghan, 2013)<br><br>Australia	Review article	This article provides a definition, description and critique of pre-loss care in music therapy.	Carers of children and adults who received music therapy at the end-of-life.	Music therapists in palliative care offer pre-loss support by conducting sessions for patients and families to convey meaningful messages, such as affirmation, grief, and reminiscences that celebrate shared experiences. Additionally, they facilitate pre-loss care by assisting patients, either independently or with their families, in crafting tangible items that honour their lives or convey significant thoughts and messages. These legacies, rooted in memories and music, serve as enduring reminders that offer solace to those grieving, fostering opportunities for continued connection with the departed that contribute to a more resilient adjustment to loss.
44	(Grewe, 2017)<br><br>USA	Original paper<br><br>Qualitative study	The aim was to study whether doing a life review process, in a group setting, with the aim of crafting a legacy of the soul for loved ones might alleviate existential distress for senior adults.	A total of 34 adults participated in the program.	The initiative outlined in this article equips chaplains with resources to tackle universal concerns that instil fear, especially among the elderly: mortality, solitude, and a sense of futility. Teams were motivated to embrace legacy activities as they align closely with the goals of palliative care. Constructing a legacy serves as a straightforward and efficient intervention that can be seamlessly integrated into clinical practice. Research suggests that incorporating a spiritual testament or a legacy of the soul into end-of-life preparations can alleviate existential distress as one approaches life's conclusion. Developing a legacy can aid in addressing both physical and existential symptoms effectively.
45	(McDermott, 2019)<br><br>Canada	Original paper<br><br>Quantitative study	To investigate the feasibility of using the Patient Dignity Question.	19 patients who met the research criteria and who were admitted to the hospice from September 2015 to December 2016.	The Patient Dignity Question presents a dignified intervention that acts as a significant end-of-life document benefiting patients, staff, and families alike. Implementing this question in the hospice enabled patients to feel genuinely listened to, while enhancing caregivers' empathy and comprehension of patients' requirements.
46	(Saracino et. al. 2019)<br><br>Canada	Review article	This paper pretends to review the empirical study of psychological well-being and distress at the end of life.	Studies of patient desire for hastened death and the early debates around physician assisted suicide.	Patients facing an uncertain future – whether through Dignity Therapy, Meaning-Centered Psychotherapy, or other interventions rooted in existential concepts – deserve and need cutting-edge interventions. The primary objective of alleviating suffering and aiding patients and their families in upholding a sense of purpose, dignity, and serenity lies at the core of these endeavours.

47	(Larson, 2005)<br><br>USA	Discussion paper	Intends to explore the main learnings and positions arising from author's professional activity.	10 lessons that were evident to the author, based on 25 years of experience as an end-of-life researcher and clinician.	The paper emphasizes the significance of stress management, effective communication, the personalized interpretation of a meaningful end-of-life experience, the crucial role of patient-centered care, the influence of self-efficacy, the importance of blending theory with practical knowledge, the value of interdisciplinary collaboration, the effects of altruism and finding purpose, the importance of attentive listening, and the therapeutic benefits of discussing grief and loss.
48	(Ho et al., 2017)<br><br>Singapore	Original paper<br><br>Quantitative study	The aims of this study are to assess the feasibility, acceptability and potential effectiveness of a Family Dignity Intervention in reducing psychosocial, emotional, spiritual and psychophysiological distress.	126 Asian families in Singapore, including participants of Chinese, Malay, Indian and other Eurasian ethnicities.	This groundbreaking study pioneers the creation and evaluation of an evidence-based, family-centered psycho-social-spiritual intervention aimed at promoting dignity and well-being for Asian patients and their families navigating end-of-life challenges. It fills a crucial void in the delivery of comprehensive palliative care. The anticipated results will drive progress in both palliative care theories and practices for Singapore and its surrounding areas, serving as a model for similar initiatives in other Asian societies.
49	(Mok et al., 2012)<br><br>China	Original article<br><br>Quantitative study	To develop a Meaning of Life Intervention in response to the need for brief and meaning focused interventions in palliative care and to establish the potential effect of the intervention.	84 adult patients with advanced-stage cancer participated in the program. Fifty-eight completed the study.	The intervention (making legacy product) was composed by two sessions during a two- to three-day window based on the sources of meaning of life proposed in logotherapy: creative, experiential, and attitudinal values. The results of RCT showed that the proposed intervention had a medium effect on Quality of Life (measured by the Quality-of-Life Concerns in the End-of-Life [QOLC-E]), namely in subscale "existential distress". Socially, participants felt that there was someone who was concerned about them. Emotionally and psychologically, the intervention induced a sense of relief. Spiritually, the intervention clarified life views and enhanced self-understanding.
50	(Duggleby and Wright, 2004)<br><br>Canada	Original paper<br><br>Qualitative study	The aim of the study was to describe the perceptions of strategies to promote hope among elderly patients with advanced cancer receiving palliative care at home.	10 participants (five males and five females).	The results enhance our comprehension of how elderly cancer patients in palliative care nurture their sense of hope. Additionally, these findings lay the groundwork for crafting successful strategies to cultivate hope within this demographic. Establishing efficient methods to foster hope will aid healthcare providers and patients' families in supporting palliative care patients to embrace hope, and ultimately pass on with dignity, tranquillity, and solace.
51	(Ando et al., 2011)<br><br>Japan	Original paper<br><br>Qualitative analysis	This study aims to identify the factors associated with improved spiritual well-being in bereaved families.	21 members of bereaved families who had lost a family member were treated in palliative care units in Japan.	Family members participated in the Bereavement Life Review across two sessions. During the initial session, they reflected on memories shared with their loved ones and assembled an album. In the subsequent session, the family member and psychologist validated the album's contents. Results indicated that aspects such as "cherished family memories," "grieving and rebuilding," and "positive recollections of final days" were linked to enhanced spiritual well-being.
52	(Chung et al., 2018)<br><br>China	Original paper<br><br>Qualitative study	The study aims to understand the experience of Chinese spouses with their loved ones who are at the end of their lives and suffering from a terminal illness.	Fifteen people took part in individual interviews (7 men and 8 women).	The study indicates that the ability to uphold relationships and derive significance during one's final days extends beyond the medical aspects of dying. Therefore, healthcare professionals could explore the potential within spouses, who may possess a natural aptitude in their relationships to derive meaning from their challenges (possibly more frequently than the patients' children). The study's outcomes emphasize the importance of implementing psychological interventions to enhance the resilience of spouses of terminally ill individuals and underscore the significance of beneficial strategies during the shift from curative to palliative care.

53	(Thompson et al. 2019)<br><br>Australia	Original paper<br><br>Qualitative study	The study seeks to comprehend the perspectives and experiences of Aboriginal individuals at the end of life.	10 Aboriginal participants.	In a secure environment, Aboriginal individuals expressed a willingness to discuss their end-of-life preferences, although some areas related to death are sensitive topics. Methods such as sorting cards, ceremonies, educational initiatives, and involving Aboriginal individuals in care roles present promising avenues to engage them effectively in end-of-life planning and navigating the grieving process. By this way person expresses matters important to them regarding their legacy, beliefs and achievements.
54	(Hovland and Kramer, 2019)<br><br>USA	Original paper<br><br>Qualitative study	This study's purpose was to explore how caregivers handle these dementia deaths.	36 caregivers of family members aged 65 and older who died from a dementia-related diagnosis.	Caregivers highlighted how they honoured the generations before them through their caregiving and how they care was modelled by legacy left by the ancestors. Creating a legacy is recognized as a facilitator to preparing caregivers for the death of a family member with dementia. The results could provide social workers and healthcare practitioners with preliminary guidance on effectively addressing caregivers' anticipation of death. By delving into continued caregiver studies, professionals can better navigate the challenges of assisting family members that are caring for individuals with dementia, particularly in coping with uncertainties surrounding the timing and preparedness for the eventual passing.
55	(Shu, 2023)<br><br>USA	Case study	Aims to describe the spiritual care relationship between an African American man receiving palliative care for metastatic cancer and a Chinese American woman chaplain over the period of multiple hospitalizations.	An African American man receiving palliative care for metastatic cancer and a Chinese American woman chaplain.	This case posits the importance of voices of chaplains of colour and encourages all chaplains to develop caregiving capacities that address patients' needs for racial justice, meaning, and spiritual legacy.
56	(Bernat et al., 2015)<br><br>USA	Original paper<br><br>Quantitative study	Aims to present a pilot abbreviated dignity therapy intervention using a legacy-building web portal for adults with terminal cancer.	Sixteen participants enrolled in the study (12 women and 4 men).	The findings indicate that the legacy-building intervention proved to be feasible and largely well-received. Participants expressed high satisfaction with the condensed intervention and the resultant legacy projects they developed, though they generally expressed dissatisfaction with the web portal.
57	(Hesse et al., 2019)<br><br>Germany	Original paper<br><br>Qualitative study	To find priorities and primary concerns of patients with a life-limiting disease who participated in a reminiscence and legacy intervention.	Seventeen patients who were receiving palliative care.	The intervention was well-received by patients, who reported feeling satisfied and experiencing a sense of well-being. The interviews revealed significant themes related to the variables that contribute to the formation and manifestation of personality, including influences that shape character, self-perception, self-awareness, and life philosophy.
58	(Collins, 2019)<br><br>Canada	Original paper<br><br>Qualitative study	To explore the impact experienced by volunteers who facilitate the legacy generation session.	5 volunteers who assisted palliative care patients with legacy creation.	Five themes emerged from the data: "providing a benefit", "internal validation", "it's all been positive", "self-awareness", and "if you need support".
59	(Stanley et al., 2023)<br><br>UK	Original paper<br><br>Qualitative study	To understand healthcare professionals' experiences of managing digital legacy.	10 palliative care healthcare professionals.	Main themes: 'accessing digital legacy'; 'becoming part of advance care planning'; 'impacting grief and bereavement'; and 'raising awareness of digital legacy'

60	(Dönmez and Johnston, 2020)<br><br>UK	Review article	To investigate the concept of 'living in the moment' within the realm of dignity-conserving care towards the end-of-life.	37 papers reviewed.	A comprehensive definition and conceptual framework of the core concept were established, outlining the theoretical interconnections between its precursors, characteristics, and outcomes. This definition and conceptual model can facilitate the creation of tools to evaluate the impacts, presence, or qualities of the concept, establish a theoretical framework, and generate new insights and approaches for nurses to enhance dignified person-centered care at the end-of-life.
